# Switching from a Fixed Monthly Aflibercept Regimen to Bi-Monthly Brolucizumab in Refractory Cases of Neovascular Age-Related Macular Degeneration

**DOI:** 10.3390/jcm13123434

**Published:** 2024-06-12

**Authors:** Minhee Kim, Ji Eon Kang, Young Gun Park

**Affiliations:** 1 Department of Ophthalmology and Visual Science, Seoul St. Mary’s Hospital, College of Medicine, The Catholic University of Korea, Seoul 06591, Republic of Korea; 2 Catholic Institute for Visual Science, College of Medicine, The Catholic University of Korea, Seoul 06591, Republic of Korea

**Keywords:** brolucizumab, bi-monthly, refractory, age-related macular degeneration, aflibercept

## Abstract

**Background/Objectives**: This study aimed to assess the effectiveness of bi-monthly brolucimumab treatment in patients with neovascular age-related macular degeneration (nAMD) refractory to monthly aflibercept treatment. **Methods**: A retrospective chart review included 32 eyes of patients with refractory nAMD who switched from monthly intravitreal aflibercept treatment to bi-monthly intravitreal brolucizumab treatment. This study evaluated changes in visual acuity (VA), intraretinal fluid (IRF), subretinal fluid (SRF), pigment epithelial detachment (PED), and central macular thickness (CMT), at specific times as follows: baseline before switching (T0), 2 months after switching (T1), 4 months after switching (T2), and 6 months after switching (T3). **Results**: The mean best-corrected visual acuity (BCVA) did not significantly change across all time points (0.52 ± 0.12, 0.48 ± 0.27, 0.48 ± 0.28, and 0.50 ± 0.27 logarithms of the minimum angle of resolution in T0, T1, T2, and T3, respectively). CMT significantly decreased after additional brolucizumab injections compared to the baseline (218.2 ± 48.6 and 207.9 ± 49.8 μm, respectively; *p* = 0.001). The PED height also significantly decreased from 251.0 ± 165.4 to 154.4 ± 115.65 μm (*p* < 0.001), with complete resolution in nine patients (28%). The mean subfoveal choroidal thickness (SFCT) before brolucizumab treatment was 262.8 ± 79.7 μm, which decreased to 233.0 ± 71.2 μm (*p* = 0.001) after the first injection. The final SFCT also significantly decreased after additional brolucizumab injections compared to the baseline SFCT (*p* = 0.012). **Conclusions**: Bi-monthly brolucizumab treatment proves effective for patients refractory to monthly fixed aflibercept, resulting in positive anatomical changes without significant deterioration in visual acuity. This approach provides a promising prognosis while reducing the treatment burden on refractory patients.

## 1. Introduction

Age-related macular degeneration (AMD) is a prominent cause of global visual impairment [1,2]. Vascular endothelial growth factor (VEGF) is known to play an important role in the regulation of neovascularization and vascular permeability [3]. Anti-VEGF agents are the primary approach for treating neovascular AMD (nAMD) [4,5]. Although the need for long-term continuous treatment is a burden, the use of treat-and-extend (T&E: shortened and extended dosing intervals for active and inactive diseases, respectively) regimens reduces patient visits and administrations compared to fixed dosing and maintains visual outcomes compared to pro re nata (PRN; dosing at exacerbations) [6].

The burden of expensive anti-VEGF agents causes poor compliance and induced under-treatment, which eventually leads to a risk of visual deterioration [7,8,9]. Therefore, long-lasting anti-VEGF agents are needed. Numerous studies have focused on evaluating the effectiveness and cost of using this therapeutic approach. The endpoint of these studies is to reduce the number and frequency of injections, thereby alleviating the pharmacological burden and costs [10]. Aflibercept (Eylea^®^; Bayer Healthcare, Berlin, Germany), a recombinant fusion protein that binds to VEGF family members, is widely used and effective in patients with AMD. However, some patients exhibit a lack of response to aflibercept during the maintenance phase despite an initial positive response during the loading phase, known as tachyphylaxis. Even if patients are treated regularly, some patients remain persistent in any compartment fluid over time. Whether this phenomenon is due to a diverse pathophysiological mechanism that is not controlled by recent anti-VEGF antibodies, such as tachyphylaxis, is not fully understood. Resistance to treatment may occur at any time during therapy in some patients. To support this, recent studies have shown evidence of tachyphylaxis for aflibercept as well as ranibizumab. In the VIEW1/VIEW2 trials, active exudation persisted in approximately 19.7% and 36.6% of the patients who received aflibercept treatment every 4 and 8 weeks for 1 year, respectively [11]. Therefore, brolucizumab (Beovu^®^; Novartis, Basel, Switzerland) was recently introduced as a new anti-VEGF agent to address the need for more effective and durable drugs.

Brolucizumab is a single-chain antibody with a molecular weight of approximately 26 kDa. Since it is a smaller molecule than other anti-VEGF drugs, a deeper penetrance through retinal tissues, tolerability, and durability are expected to be efficient against nAMD [12,13]. The pivotal phase III clinical trials HAWK and HARRIER studies showed the non-inferiority of visual function in patients with treatment-naïve nAMD treated with brolucizumab when compared to those treated with aflibercept [14,15]. The SHIFT study showed significant outcomes in the switch to brolucizumab, suggesting it to be a good option for non-responsive nAMD [16]. Although a switch from aflibercept to intravitreal brolucizumab injection may be efficacious in patients with nAMD, only a few reports have demonstrated the efficacy of such a switch and treatment intervals [17,18]. Notably, considerable effort is underway to minimize injections and enhance efficacy in nAMD treatment. Recent studies have shown the efficacy of switching to brolucizumab for patients with refractory nAMD compared to previous anti-VEGF treatments [19,20]. However, when patients with nAMD switch anti-VEGF agents, three monthly intravitreal injections are recommended to start as a loading dose regimen even if the previously maintained administration interval had been used before switching. Therefore, previous studies are limited to these loading regimens.

This study aimed to assess the visual and anatomical outcomes resulting from switching patients with refractive nAMD from monthly aflibercept to bi-monthly brolucizumab without a loading dose regimen in a real-world setting. As recent advancements and challenges in AMD treatment continue to shape the field, this study contributes to the ongoing discourse, providing insights into optimizing treatment strategies and minimizing patient burden.

## 2. Materials and Methods

### 2.1. Ethics Statements

This study was conducted according to the guidelines of the Declaration of Helsinki (1964). This retrospective study was conducted using data from medical records. Approval was obtained from the Institutional Review Board/Ethics Committee of Seoul St. Mary’s Hospital, Catholic University of Korea (approval number: KC23RASI0921). The Institutional Review Board waived the need for informed consent because of the retrospective nature of the study.

### 2.2. Study Design and Patients

This retrospective study focused on consecutive patients who visited the Department of Ophthalmology of Seoul St. Mary’s Hospital in Seoul, Republic of Korea, between January 2022 and November 2023. The patients had refractory nAMD and had received monthly aflibercept injections within the past 6 months. Inclusion criteria considered patients to be refractory if they required aflibercept due to persistent or recurrent fluid, such as subretinal fluid (SRF), intraretinal fluid (IRF), or subretinal pigment epithelium (RPE) (sub-RPE) fluid despite at least six monthly aflibercept injections. Exclusion criteria included active subretinal hemorrhage, geographic atrophy, polypoidal choroidal vasculopathy (PCV), retinal angiomatous proliferation, and secondary macular neovascularization (MNV) due to other causes.

### 2.3. Treatment Protocol

A T&E protocol is typically initiated with ≥3 consecutive monthly injections until disease inactivity is established, followed by a gradual extension of the treatment interval in increments of 2–4 weeks, up to a maximum interval of 12–16 weeks. Treatment intervals are shortened when disease activity recurs. Anti-VEGF injections are given at every scheduled visit despite disease inactivity [21,22].

In our study, patients previously received three monthly loading injections of aflibercept before switching, followed by a T&E protocol. Patients who had an insufficient response to aflibercept and reduced treatment interval to monthly were treated with at least six monthly injections and were included in this study. The decision to switch from aflibercept to brolucizumab was determined by the physician when patients met the following insufficient response conditions: if the patients had persistent SRF, IRF, and/or sub-RPE fluid on swept-source optical coherence tomography (OCT) (SS-OCT), compared to the baseline for at least six monthly intravitreal aflibercept injections. After that, the patients switched to brolucizumab at bi-monthly intervals three times.

### 2.4. Intervention and Procedure

Patients underwent standardized dilated fundus examinations, including best-corrected visual acuity (BCVA) measurements, using wide-field fundus photography (Optos PCL, Scotland, UK) and SS-OCT (DRI Triton, Topcon, Japan).

We measured central macular thickness (CMT), which was defined as the distance between the vitreoretinal surface and the inner surface of the RPE. Subfoveal choroidal thickness (SFCT) was measured as the length between the outer border of Bruch’s membrane and the chorioscleral interface using enhanced depth imaging scans. CMT and SFCT were measured in horizontal and vertical scans and then averaged. Pigment epithelium detachment (PED) height was defined as the distance between the outer border of the RPE and the inner border of Bruch’s membrane. Graders reviewed all the B-scans to determine and measure the maximum PED height in the raster scans. A dry macula was defined as the complete resolution of IRF and/or SRF detected on SS-OCT raster scans. The persistence of only PED was considered to indicate a dry macula. Assessments were conducted at the baseline (T0) and then repeated at 2 months (T1), 4 months (T2), and 6 months (T3) post-treatment. Outcomes included OCT structural activity biomarkers, such as IRF and SRF variations, PED height variations, CMT, and BCVA at the baseline, T1, T2, and T3. We excluded images with a signal strength index of <50 from the analysis.

### 2.5. Statistical Analysis

BCVA values were transformed into logarithms of the minimum angle of resolution (logMAR) values. Means and standard deviations (SDs) were calculated for quantitative variables. Repeated-measures analysis of variance with Bonferroni’s correction and paired *t*-tests were employed for statistical analysis. All statistical analyses were performed using IBM SPSS Statistics for Windows, version 22.0 (IBM Corp., Armonk, NY, USA), and statistical significance was set at *p* < 0.05.

## 3. Results

### 3.1. Baseline Characteristics

This study included 32 eyes of 32 patients with nAMD. Nineteen and thirteen patients were males and females, respectively, with a mean age of 72.12 ± 8.23 (range: 59–85) years. The switched patients had received an average of 17.45 ± 8.34 (range: 9–22) years of aflibercept before switching to brolucizumab. Table 1 presents the patient characteristics.

### 3.2. Visual Outcomes after Intravitreal Brolucizumab Injection

**Figure 1:**
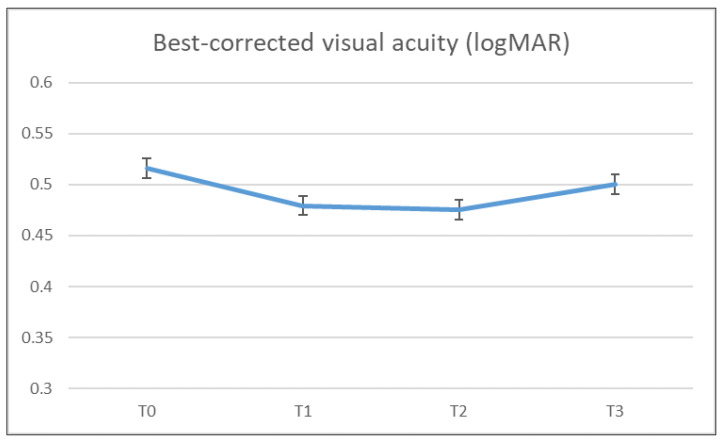
Changes in best-corrected visual acuity.

The mean BCVA did not significantly change from the baseline at the various follow-up time points (0.52 ± 0.12, 0.48 ± 0.27, 0.48 ± 0.28, and 0.50 ± 0.27 logMAR in T0, T1, T2, and T3, respectively). Vision remained stable even after additional brolucizumab treatment. Figure 1 shows the graphical representation of the changes in BCVA.

### 3.3. Optical Coherence Tomography Measurements after Intravitreal Brolucizumab Injection

#### 3.3.1. Central Macular Thickness

**Figure 2:**
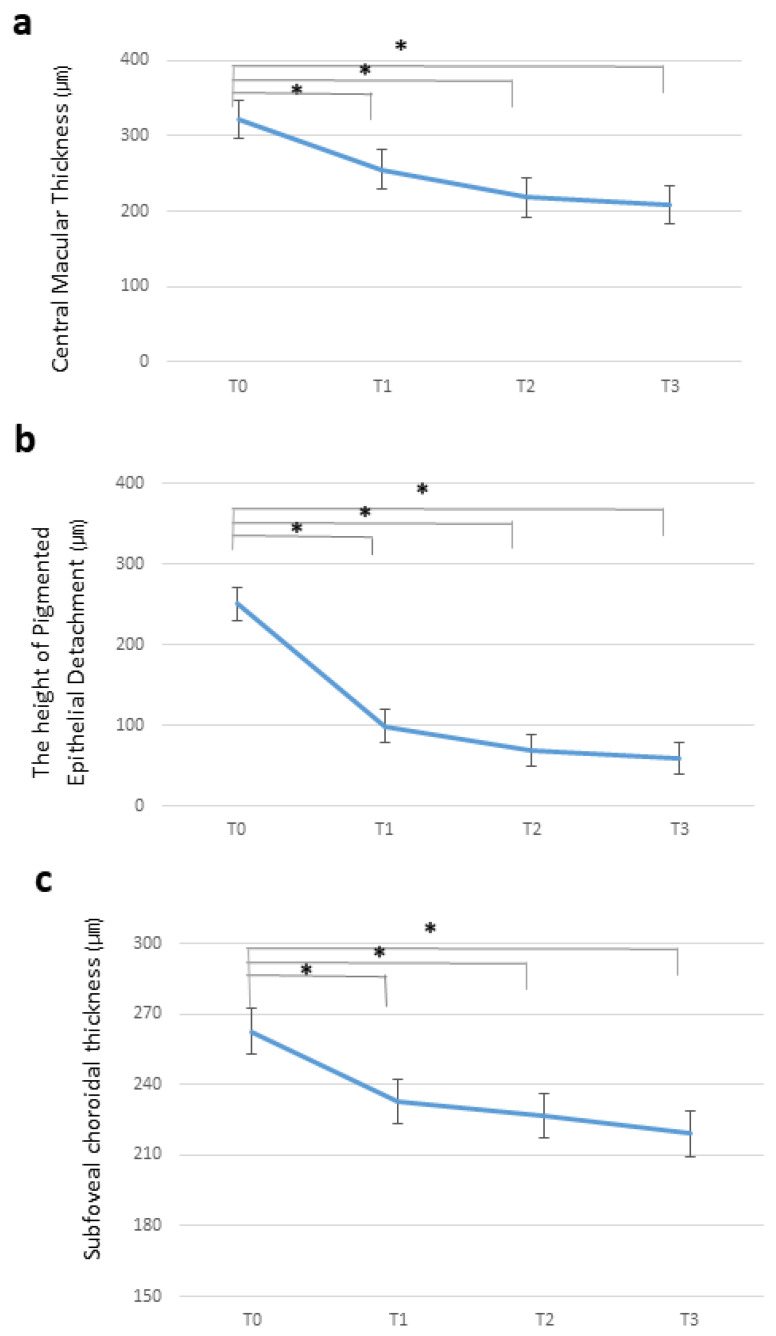
Changes in the mean central macular thickness (**a**), pigment epithelial detachment height (**b**), and subfoveal choroidal thickness (**c**) from before to after switching to brolucizumab. * statistically significant at the *p* < 0.05.

The initial CMT was 321.3 ± 91.9 μm, significantly decreasing to 255.6 ± 40.2 μm after the first brolucizumab treatment (*p* < 0.001). Additional brolucizumab injections further reduced CMT, reaching 218.2 ± 48.6 and 207.9 ± 49.8 μm after the second and third injections, respectively (*p* = 0.001). Figure 2a shows the temporal changes in CMT.

Complete resolution of IRF and SRF was observed in 13 (40.6%) eyes after a single dose of brolucizumab and 24 (75%) eyes after three doses of brolucizumab.

#### 3.3.2. Pigment Epithelial Detachment Height

A single dose of brolucizumab significantly reduced PED from 251.0 ± 165.4 to 165.7 ± 100.5 μm (*p* < 0.001). At T2 and T3, PED heights continued to decrease to 169.7 ± 103.1 and 154.4 ± 115.65 μm. At the final visit (T3), nine patients (28%) showed complete resolution of PED. Temporal changes in the PED height are shown in Figure 2b.

#### 3.3.3. Subfoveal Choroidal Thickness

Before brolucizumab treatment, the mean SFCT was 262.8 ± 79.7 μm, which decreased to 233.0 ± 71.2 μm (*p* = 0.001) after the first injection. Additional brolucizumab injections led to a further reduction in SFCT compared to the baseline, with values of 226.9 ± 68.4 and 219.1 ± 64.5 μm at T2 and T3, respectively (*p* = 0.012). Temporal changes in SFCT are shown in Figure 2c.

#### 3.3.4. Intraocular Inflammation

Among the 32 eyes, 2 (6.3%) reported adverse events during the observation period. One eye showed anterior chamber inflammation alone and was treated with topical steroid eye drops, whereas the other showed mild vitreous opacity without occlusive vasculitis and was treated with a subtenon triamcinolone injection. The patient recovered without persistent complications post-treatment.

## 4. Discussion

**Figure 3:**
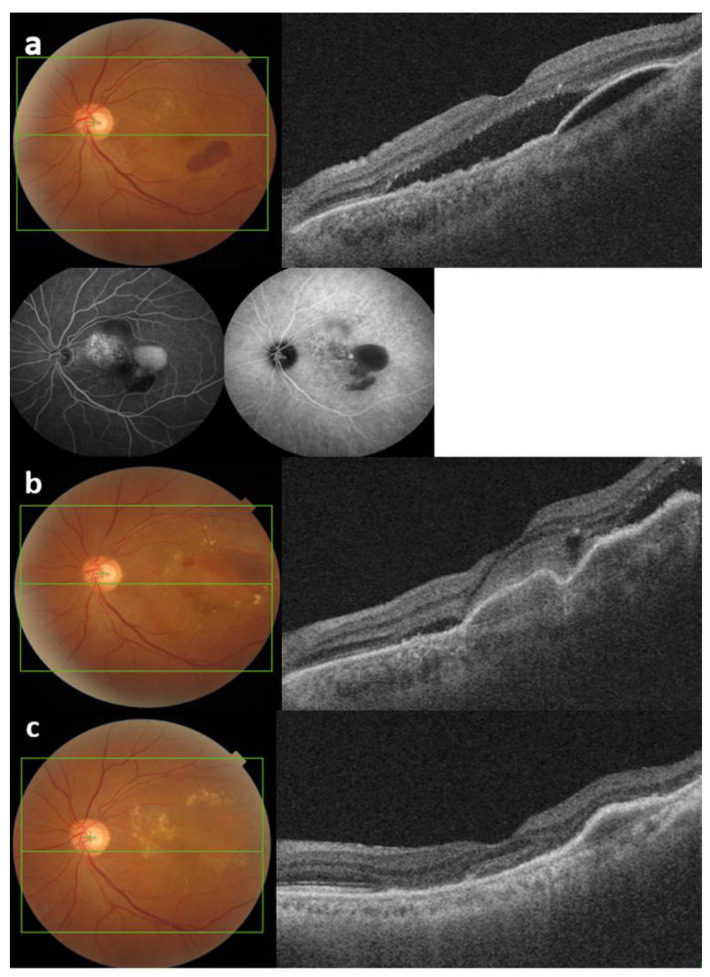
A 69-year-old male patient was initially diagnosed with type 1 macular neovascularization in the left eye. He had a best-corrected visual acuity (BCVA) of 0.49 logMAR. (**a**) Color fundus photograph and swept-source optical coherence tomography (SS-OCT) showed large pigment epithelial detachment with subretinal fluid at the initial visit. Fluorescein and indocyanine green angiographies showed leakage suggestive of occult choroidal neovascularization. (**b**) SS-OCT showed subretinal fluid (SRF) aggravation with increased subretinal hemorrhage despite eleven monthly aflibercept injections. He started receiving bi-monthly brolucizumab three times. (**c**) The SRF and hemorrhage resolved completely 6 months after the switch to brolucizumab.

**Figure 4:**
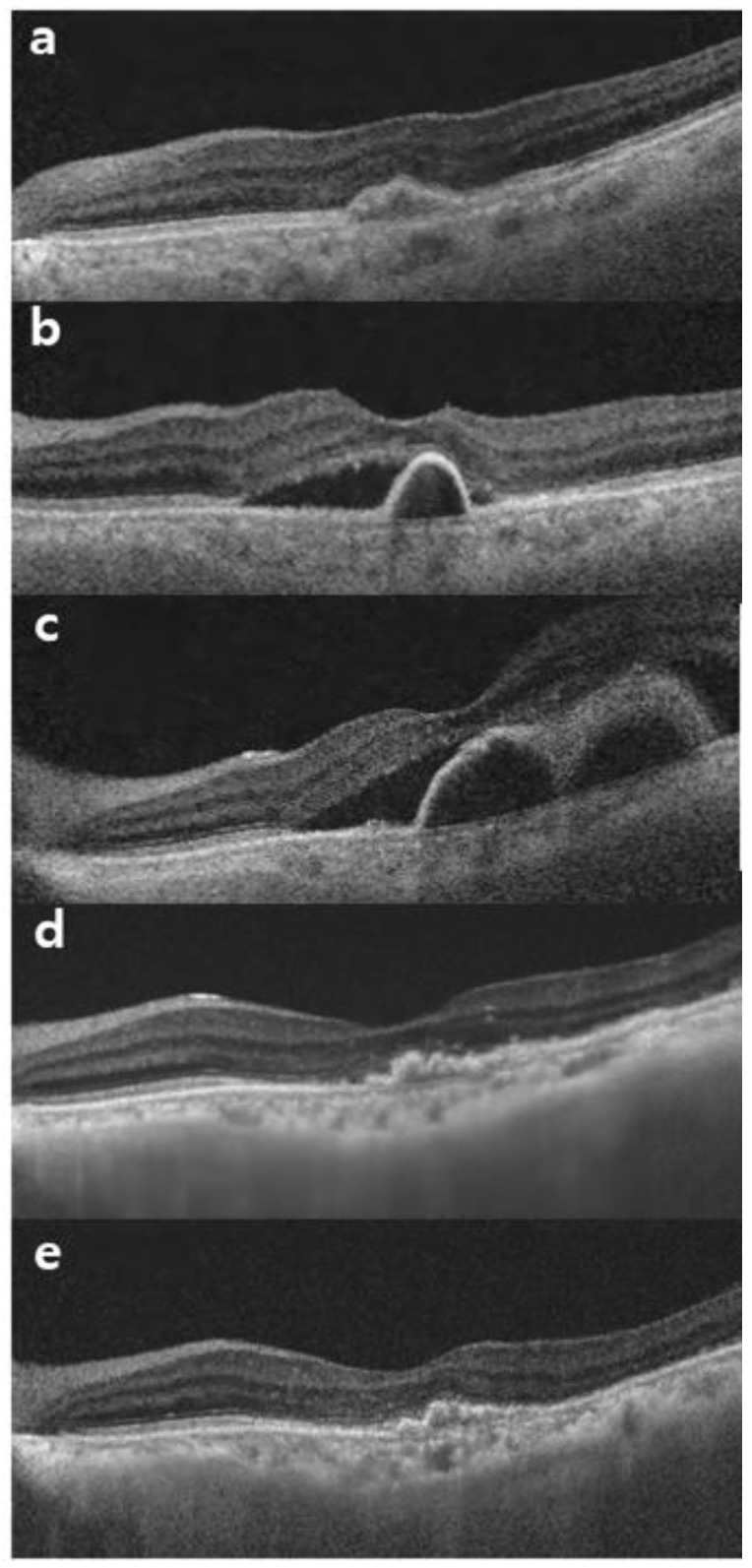
A representative case of a 73-year-old male patient with type 1 macular neovascularization in the left eye. (**a**) He has already received eight intravitreal aflibercept injections and maintained treatments. (**b**) After that, new subretinal fluid (SRF) with pigment epithelial detachment (PED) appeared. He received monthly aflibercept injections. (**c**) Despite three monthly aflibercept injections, SRF with multiple PEDs increased. (**d**) He started receiving brolucizumab injections. After switching, the SRF and PED were resolved completely. (**e**) Six months after the switch to brolucizumab, no more fluid was observed.

This is the first study to identify the success of switching to brolucizumab for aflibercept–refractory AMD with longer dosing intervals without a loading period. Switching from aflibercept to brolucizumab for refractory nAMD results in improved visual outcomes in many cases [23,24]. However, these reports are limited to three loading periods with brolucizumab. In our study, the participants received treatment for an extended period before switching (Figure 3 and Figure 4).

Tachyphylaxis is a not fully understood phenomenon, which can be defined as a poor response, after an initial successful response to the treatment. Tachyphylaxis to anti-VEGF therapy has been reported to happen at a rate of 10% for bevacizumab and a rate of 7.7% for ranibizumab [25,26]. Hara et al. demonstrated that 8.9% of treatment-naïve patients developed tachyphylaxis after intravitreal aflibercept treatment [27]. According to previous reports, switching from one type of intravitreal drug to another may be effective for eyes that develop tachyphylaxis. Switching to another anti-VEGF agent is helpful in restoring the effectiveness of a drug in patients with tachyphylaxis.

Our study establishes brolucizumab as an effective alternative to aflibercept for patients with refractive nAMD requiring an extended treatment interval. Patients switching from monthly aflibercept to bi-monthly brolucizumab experienced significant improvements in anatomical outcomes, including CMT, SFCT, and PED heights. Although no significant difference in BCVA exists, reduced injection frequency demonstrated sustained efficacy over a prolonged period. Previous studies have demonstrated the effectiveness of brolucizumab loading doses in both naïve and refractory nAMD [24,28,29]. However, these studies primarily concentrated on monthly loading methods diverging from real-world clinical practices. The absence of guidance on the bi-monthly dosing of brolucizumab without three initial loadings in refractory patients underscores the significance of our study.

Patients with nAMD usually require numerous anti-VEGF injections to manage the disease, leading to a considerable treatment burden due to frequent injections and regular monitoring [30,31]. However, ensuring the delivery of the most effective and optimized treatment for patients using new anti-VEGF agents at appropriate treatment intervals is crucial.

Brolucizumab has already been identified with long-lasting effects and strong efficacy in controlling exudative changes in the HAWK and HARRIER trials [32]. Recent reports showed short-term real-world outcomes of brolucizumab treatment for nAMD. Bulirsch et al. showed that the mean CMT was decreased by 66.8 µm compared to the baseline 1 month after switching from ranibizumab, bevacizumab, and aflibercept [16]. Our study shows that the mean CMT significantly decreased by 65.0 µm 1 month after switching from aflibercept to brolucizumab, which is similar to previous results.

Furthermore, our study specifically targeted patients with wet AMD resistant to aflibercept, defined as those with SRF or IRF despite regular monthly injections. In real-world clinical settings, the T&E regimen is commonly used after three loading doses of anti-VEGF agents. However, if the response diminishes, clinics shift from a T&E regimen to a monthly fixed-dose approach. Notably, patients in our study received over 10 aflibercept injections before switching to brolucizumab. Tamiya et al. reported that 56% of patients with aflibercept–refractory nAMD showed a reduction or complete absorption after switching to faricimab [33]. The treatment-resistant patients are at a particularly high risk of discontinuing treatment due to frustration with poor response and the need for frequent injections [34,35]. Initial positive responses usually decrease over time, which leads to increased pressure and reduced compliance, thereby resulting in adverse outcomes [36].

IRF and SRF are important markers of MNV activity in patients with nAMD [37]. Persistent or recurrent fluid accumulation despite anti-VEGF therapy leads to disease progression. In our study, complete fluid resolution after switching to a single brolucizumab injection was achieved in 40.6% of patients with persistent fluid despite multiple monthly aflibercept injections. It suggests that brolucizumab demonstrates a durable treatment response in some patients with refractory nAMD. This notable fluid reduction without monthly injections contributes to the long-term preservation of vision and improved quality of life in patients.

Our results indicated that switching to brolucizumab may be a viable option, particularly for addressing morphological effects in patients with nAMD, previously treated with multiple anti-VEGF injections without sufficient fluid resolution across various anatomical compartments. A significant reduction in CMT was observed at all time points, indicating a favorable response to morphological signs of disease activity. Correspondingly, Bulirsch et al., in the SHIFT trials, showed improved anatomical outcomes in 63 eyes of 57 patients with nAMD who switched to brolucizumab [16]. Other reports also indicated beneficial improvements in various OCT characteristics at the first visit after the switch to brolucizumab [24,38]. Matsumoto et al. observed significant BCVA improvement 1 month after the initial injection of brolucizumab in 36 eyes of patients with nAMD associated with type 1 choroidal neovascularization (CNV) [39,40]. However, our results showed no significant changes in BCVA at T1, T2, and T3 compared to the baseline, despite notable functional improvements after brolucizumab injections. This limitation can be attributed to the prolonged treatment duration in our patient group, as opposed to treating naïve individuals with nAMD.

SFCT varies among different subtypes of nAMD [41,42], and the impact of intravitreal injections of anti-VEGF agents on choroidal thickness differs. Monitoring choroidal thickness after brolucizumab injections may be important for the optimal management of nAMD. Brolucizumab is a small protein fragment of approximately 26 kDa. Because of its advantages, the penetration and concentration of brolucizumab was 42% in the central retina and 18% in the choroid relative to the vitreous [43]. Recent reports have highlighted choroidal thickness changes in patients with nAMD after brolucizumab treatment [38,44].

Matsumoto et al. observed a 15.5% decrease in choroidal thickness after three monthly brolucizumab injections in patients with treatment-naïve type 1 CNV [45]. Bae et al. also reported a significant decrease in choroidal thickness after the initial brolucizumab injection in 34 eyes [38]. While our study excluded patients with PCV due to its influence on choroidal thickness outcomes, our results indicated a significant decrease in SFCT at all time points compared to the baseline (*p* < 0.005), even in patients with refractory nAMD compared to monthly aflibercept injections. However, the implications of thinner choroids remain unclear. Koizumi et al. reported that a thinner choroid correlates with better visual outcomes after aflibercept injection [46], but some studies identify it as a risk factor for macular atrophy [47]. Therefore, further research is necessary to ascertain the long-term prognosis of choroidal changes after brolucizumab treatment.

In a real-world setting, the most important reason for the difficulty in continuing brolucizumab treatment is the brolucizumab-associated intraocular inflammation (IOI). Brolucizumab-associated IOI was reported to range from mild iritis/vitritis to severe retinal vasculitis with/without vascular occlusion and irreversible vision loss [48,49]. Although the HAWK and HARRIER trials reported higher rates of IOI with brolucizumab (4.6%) than with aflibercept, some clinical studies showed a higher incidence of IOI ranging between 13.9% and 19.0% [45,50]. In our study, the incidence of IOI is 6.3%. All cases were mild to moderate inflammation without occlusive vasculitis and resolved without complications. The pathophysiology behind the higher rate of IOI associated with brolucizumab is unclear. In our cases, the rate of IOI is not higher than that reported in other studies, and mild progression with visual recovery was observed. Therefore, we suggest the longer treatment intervals may reduce the incidence and degree of IOI responses after brolucizumab injections.

This study had some limitations, including its retrospective nature, small patient cohort, a mix of AMD types, and focus on a Korean population. However, we consecutively enrolled patients treated with brolucizumab as a switching therapy due to refractory responses, specifically those who had received prior aflibercept treatment alone. Therefore, we used real-world data from clinics that treat nAMD. In summary, intravitreal brolucizumab injections as a switching treatment significantly reduced CMT; SFCT; IRF and SRF presence; and PED heights in patients with refractory nAMD despite the extended treatment interval.

## 5. Conclusions

This study demonstrated that bi-monthly brolucizumab treatment without three monthly loading injections is a potent therapeutic approach for refractory patients compared to other monthly fixed anti-VEGF agents. We also achieved a favorable prognosis by alleviating the treatment burden on refractory patients.

## Figures and Tables

**Table 1:** Patient baseline characteristics.

Variables	Patients
Age (years)	72.12 ± 8.23
Sex, male (%)	19 (59.3%)
Number of injections before the switch	17.45 ± 8.93
Best-corrected visual acuity (logMAR)	0.52 ± 0.23
Central macular thickness (μm)	321.27 ± 91.89
Subfoveal choroidal thickness (μm)	262.76 ± 79.73
Pigment epithelial detachment height (μm)	251.00 ± 165.41

LogMAR, logarithms of the minimum angle of resolution.

## Data Availability

The datasets analyzed during the current study are available from the corresponding authors upon reasonable request.
